# Adenosine Receptors: Expression, Function and Regulation

**DOI:** 10.3390/ijms15022024

**Published:** 2014-01-28

**Authors:** Sandeep Sheth, Rafael Brito, Debashree Mukherjea, Leonard P. Rybak, Vickram Ramkumar

**Affiliations:** 1 Department of Pharmacology and Neuroscience, Southern Illinois University School of Medicine, Springfield, IL 62702, USA; E-Mails: ssheth@siumed.edu (S.S.); vramkumar@siumed.edu (V.R.); 2 Department of Surgery (Otolaryngology), Southern Illinois University School of Medicine, Springfield, IL 62702, USA; E-Mails: dmukherjea@siumed.edu (D.M.); lrybak@siumed.edu (L.P.R.); 3 Department of Neurobiology and Program of Neurosciences, Institute of Biology, Fluminense Federal University, Niteroi, RJ 24020141, Brazil; E-Mail: rafaelbs2002@yahoo.com.br

**Keywords:** adenosine, adenosine receptors, desensitization, nuclear factor-κB, sleep, hearing loss, cancer

## Abstract

Adenosine receptors (ARs) comprise a group of G protein-coupled receptors (GPCR) which mediate the physiological actions of adenosine. To date, four AR subtypes have been cloned and identified in different tissues. These receptors have distinct localization, signal transduction pathways and different means of regulation upon exposure to agonists. This review will describe the biochemical characteristics and signaling cascade associated with each receptor and provide insight into how these receptors are regulated in response to agonists. A key property of some of these receptors is their ability to serve as sensors of cellular oxidative stress, which is transmitted by transcription factors, such as nuclear factor (NF)-κB, to regulate the expression of ARs. Recent observations of oligomerization of these receptors into homo- and heterodimers will be discussed. In addition, the importance of these receptors in the regulation of normal and pathological processes such as sleep, the development of cancers and in protection against hearing loss will be examined.

## 1. Introduction

Adenosine is produced primarily from the metabolism of adenosine triphosphate (ATP) and exerts pleiotropic functions throughout the body. In the central nervous system (CNS), adenosine plays important functions such as modulation of neurotransmitter release [1], synaptic plasticity [2] and neuroprotection in ischemic, hypoxic and oxidative stress events [3–5]. In addition, adenosine plays different roles in a large variety of tissues. In the cardiovascular system, adenosine produces either vasoconstriction or vasodilation of veins and arteries [6]. Adenosine regulates T cell proliferation and cytokine production [7]. The nucleoside also inhibits lipolysis and stimulates bronchoconstriction [8,9].

Adenosine is produced both intracellularly and extracellularly. Intracellular adenosine is produced from its immediate precursor, 5′-adenosine monophosphate (5′-AMP), by the action of the enzyme 5′-nucleotidase. Adenosine can then follow several metabolic/synthetic pathways. It can be metabolized to inosine and hypoxanthine by adenosine deaminase and to uric acid by xanthine oxidase. Adenosine can also be transported out of the cell to the extracellular space by specific bi-directional nucleoside transporters [10]. Inhibitors of these transporters, such as dipyridamole, increase the extracellular concentrations of adenosine and are useful clinically to treat certain cardiovascular complications. Adenosine can also be converted back to 5′-AMP by the action of the enzyme adenosine kinase and subsequently to ADP and ATP. The extracellular 5′-AMP is produced by degradation of ATP (by ecto-nucleotidase) and cyclic AMP (by ecto-cyclic AMP phosphodiesterase) [10,11].

Unlike vesicular release of neurotransmitters upon electrical impulse, the release of adenosine is mediated mainly by the action of its transporters. Adenosine transporters are classified into equilibrative nucleoside transporters (ENTs) and concentrative nucleoside transporters (CNTs) [12]. ENTs are passive bidirectional transporters which transport adenosine across the plasma membrane based on its concentration gradients. The human genome encodes four ENT isoforms (ENT1-4). Among them, the best studied are ENT1 and ENT2 which are broadly classified depending on their sensitivity for inhibition by nitrobenzylthioinosine (NBMPR) [13]. These transporters are widely expressed in the CNS, with ENT1 showing the highest expression [14]. The CNTs are active Na^+^-dependent transporters that transport adenosine against its concentration gradient [5,15]. CNTs have been described in brain macrophages, thymocytes, liver, lung, choroid plexus, kidneys and intestines of animals [16].

Adenosine initiates its biological effects via four receptor subtypes, namely the A_1_, A_2A_, A_2B_ and A_3_ARs. The A_1_ and A_2A_AR possess high affinity for adenosine while the A_2B_ and A_3_AR show relatively lower affinity for adenosine receptors [5]. These receptors belong to the superfamily of G protein-coupled receptors (GPCR). Members of this family have a very similar molecular architecture. They display a seven transmembrane α-helical structure, with an extracellular amino-terminus and an intracellular carboxy-terminus. The *N*-terminal domain has *N*-glycosylation sites which influences trafficking of the receptor to the plasma membrane [17,18]. The carboxy-terminus contains serine and threonine residues which serve as phosphorylation sites for protein kinases and enable receptor desensitization. Furthermore, the carboxy-terminus and the third intracellular loop enable coupling of the ARs to G proteins [17,18].

ARs have traditionally been classified based on their differential coupling to adenylyl cyclase to regulate cyclic AMP levels. The A_1_ and A_3_ARs are coupled to G_i/o_ proteins, while A_2A_AR and A_2B_AR are coupled to G_s/olf_ proteins [5]. Therefore, activation of the A_2A_ and A_2B_ARs increase cyclic AMP production, resulting in activation of protein kinase A (PKA) and phosphorylation of the cyclic AMP response element binding protein (CREB). In contrast, activation of the A_1_ and A_3_AR inhibits cyclic AMP production and decreases PKA activity and CREB phosphorylation [3,5,12]. In some cases, the A_1_AR increases phospholipase C (PLC) activity through a pertussis toxin-sensitive G protein. The A_1_AR can directly couple to and inhibit cardiac K^+^ channels and types Q, N and P voltage sensitive Ca^2+^ channels. In turn, the A_3_AR can regulate the activity of PLC via a pertussis toxin-sensitive G protein in rat basophilic leukemia cells [19,20] or by direct coupling to G_q_ protein [5,21]. Activation of the A_1_AR can also increase mitogen-activated protein kinase (MAPK) pathway in cells of the Chinese hamster ovary (CHO) [22,23] and COS-7 fibroblast-like cells by βγ subunit of G_i/o_ protein [24]. Activation of the A_2A_AR can also promote activation of protein kinase C (PKC) in a cyclic AMP-dependent and independent mechanisms [5,25]. In contrast, activation of the A_2B_AR can stimulate PKC activity by direct coupling to G_q_ proteins [5].

In the CNS, A_1_ARs are widely distributed on neurons in the cortex, hippocampus and cerebellum [5]. These receptors are also present on astrocytes [26], oligodendrocytes [27] and microglia [28]. In neurons, A_1_AR are highly localized to synaptic regions, where they modulate the release of neurotransmitters, such as glutamate, acetylcholine, serotonin and GABA [3]. A_2A_AR have a more restrictive localization in the striatum and olfactory bulb [5]. These receptors are present in neurons, microglia and oligodendrocytes and possibly astrocytes [29,30]. The presence of A_2A_AR has been described in dendritic spines and postsynaptic regions of the basal ganglia [31]. These receptors are highly localized to presynaptic regions (in hippocampus), where they modulate the release of neurotransmitters such as glutamate, acetylcholine, GABA and noradrenaline [32–35]. A_2B_AR are expressed at low levels on neuronal and glial cells, such as microglia and astrocytes [36,37]. Low levels of the A_3_AR are detectable in the hippocampus, cortex, cerebellum and striatum [5], with cellular localization in neurons, astrocytes and microgila [38–40].

## 2. Desensitization of Adenosine Receptors

Continued or repeated exposures to an agonist usually result in the receptor-mediated response to plateau and then diminish despite the continual presence of agonist. In the field of receptor kinetics, this process is called desensitization [41]. Desensitization of GPCRs is a complex phenomenon involving multiple and temporally distinct components. The mechanisms underlying rapid desensitization often involve receptor phosphorylation by a family of G protein-coupled receptor kinases (GRKs) resulting in their preferential binding to arrestins molecules. This promotes desensitization by uncoupling the receptor from G-protein, leading to impaired receptor function [42]. Following desensitization, many GPCRs internalize by an arrestin-dependent process via clathrin-coated pits which leads to the eventual intracellular dephosphorylation of the receptor, and its re-insertion into the cell membrane to produce a resensitized state [43]. More prolonged agonist activation generally leads to the transfer of internalized receptor to a lysosomal compartment with subsequent down-regulation [44]. The process of desensitization, internalization, resensitization, or down-regulation forms an important part in receptor regulation which eventually controls receptor-mediated signaling pathways.

It has been shown that activation of all four AR subtypes eventually leads to their desensitization by different mechanisms. Most of these observations are made following administration of non-hydrolyzable agonists to cell cultures and animals. However, it is yet unclear how significant a role receptor desensitization plays in regulation of ARs by adenosine *in vivo* under normal conditions. This process should become more important with the use of selective AR subtype agonists for the treatment of diseases. Studies have shown that upon addition of agonists, the A_1_AR is phosphorylated and internalized slowly, with a half-life of several hours. In contrast, A_2A_AR and A_2B_AR demonstrate a more rapid rate of down-regulation, usually lasting about an hour. The down-regulation and desensitization of the A_3_AR occurs within minutes [45]. Thus, it is important to understand their regulation in order to design drugs that can exploit or avoid the receptor-mediated signaling to treat diseases.

### 2.1. A_1_AR

Several studies have been carried out to understand the molecular mechanisms underlying A_1_AR desensitization. In an early study, Parson and Stiles [46] showed that the A_1_AR in rat adipocytes desensitized upon chronic administration of A_1_AR agonist, R-phenylisopropyladenosine (R-PIA), over a period of six days by Alzet minipumps (Alza Corporation, Vacaville, CA, USA). A decrease in A_1_AR levels and reduced inhibition of isoproterenol-stimulated adenylyl cyclase activity was detected following R-PIA treatment. These changes were associated with decreased levels of pertussis-sensitive G proteins, but an increase in (cholera toxin-labeled) G_s_ proteins in the plasma membranes. Furthermore, adipocyte membranes obtained from R-PIA-treated rats showed enhance isoproterenol and forskolin-stimulated adenylyl cyclase. These studies suggest the response of adipocytes to chronic activation of the A_1_AR (*i.e*., loss of inhibition of adenylyl cyclase) is to increase the stimulatory response (*i.e*., increase in adenylyl cyclase activity). Alternatively, desensitization of the A_1_AR could relieve a tonic inhibitory tone which is reflected by enhanced agonist stimulation of adenylyl cyclase. A subsequent study performed in cultured adipocytes confirmed the *in vivo* findings and further demonstrated that desensitization of the A_1_AR was associated with desensitization of insulin-dependent glucose transport [47]. This suggests a common link between desensitization of the A_1_AR and insulin receptor. A later study by Longabaugh *et al.* [48] confirmed the previous findings that desensitization of the A_1_AR to R-PIA was linked to reductions in A_1_AR and G_i_ proteins and an increase in G_sα_ proteins, but showed that the changes in G_iα_ proteins were not linked to alternations in their mRNA levels.

Studies in a clonal cell line, ductus deferens smooth muscle (DDT_1_-MF2 cells), showed differential rates of desensitization of the A_1_AR and A_2A_AR, which were not associated with changes in their coupling to G proteins. Increased phosphorylation of the A_1_AR was observed following exposure of these cells to agonists [49]. However, the mechanism(s) underlying phosphorylation was not determined. This study demonstrates that homologous desensitization of the A_1_AR was associated with phosphorylation and uncoupling of the receptor from its G_i_ protein. Similarly, Nie *et al.* [50] demonstrated rapid translocation of GRK from cytosol to the plasma membrane and subsequent phosphorylation of the A_1_AR within one hour of R*-*PIA treatment to DDT_1_-MF2 cells. Furthermore, purified preparations of the A_1_AR that were phosphorylated with purified recombinant GRK-2 displayed enhanced affinity for arrestin over G_i_/G_o_ G-proteins. In contrast to these findings, short-term exposure of CHO cells, stably expressing human A_1_AR, to R-PIA resulted in neither desensitization nor phosphorylation by GRK [51]. The reason for these different findings is not clear at present, but could reflect differences between hamster and human A_1_AR used in these studies. An interesting finding in DDT_1_-MF2 cells was that ectoenzyme adenosine deaminase, was shown to play an important role in agonist-induced A_1_AR regulation by enhancing desensitization and internalization in DDT_1_-MF2 cells after chronic exposure to R*-*PIA. It was demonstrated that upon exposure to the agonist, adenosine deaminase and A_1_AR colocalize and internalize by the same endocytotic pathway [52].

In primary cultures of cerebellar granule cells, chronic treatment with A_1_AR agonist and antagonist reciprocally regulates A_1_AR [53]. Exposure to A_1_AR agonist, *N*^6^-cyclopentyladenosine, resulted in time- and concentration-dependent reduction in the density of the A_1_AR and G-protein coupling corresponding to impaired agonist-induced adenylyl cyclase inhibition.

Coelho *et al.* [54] reported that hypoxia decreases the density of A_1_AR in rat hippocampal slices. This desensitization could be mimicked by 2-chloroadenosine (CADO), and was prevented by adding the A_1_AR antagonist, 1,3-dipropyl-8-cyclopentylxanthine (DPCPX). These results suggest that hypoxia leads to an increase in extracellular adenosine levels, and a subsequent, quite rapid (<90 min) desensitization of A_1_AR.

Jajoo *et al.* [55] examined the role of β-arrestin1/extracellular signal-regulated kinase (ERK1/2) MAPK pathway in the regulation of A_1_AR desensitization and recovery in DDT_1_-MF2 cells. They reported that the exposure of A_1_AR agonist, R-PIA, for 24 h resulted in a decrease of A_1_AR membrane protein which was associated with an unexpected 11-fold increase in A_1_AR mRNA. This effect of R-PIA was dependent on β-arrestin1, as knockdown of β-arrestin1 by siRNA blocked R-PIA-mediated down-regulation of the A_1_AR. In addition, β-arrestin1 knockdown by siRNA suppressed R-PIA-dependent ERK1/2 and activator protein-1 (AP-1) activities and reduced the induction of A_1_AR mRNA. Interestingly, withdrawal of the agonist after a 24 h exposure resulted in rapid recovery of plasma membrane A_1_AR, which was dependent on the *de novo* protein synthesis and on the activity of ERK1/2 but independent of β-arrestin1 and NF-κB. These findings suggest that the β-arrestin1/ERK1/2 pathway, which contributes to the desensitization and down-regulation of A_1_AR membrane protein, is also able to prime the transcriptional machinery for rapid synthesis of the A_1_AR upon withdrawal of the agonist.

Chick embryo retina cultures of eight days treated for 48 h with CGS 21680 and *N*6-(2(3,5-dimethoxyphenyl)-2-(2-methylphenyl)ethyl)-adenosine (DPMA), selective A_2A_AR agonists, showed an increased expression of A_1_AR. This effect was blocked by the selective A_2A_AR antagonist, ZM 241385, and PKA inhibitor, H89 [56]. These *in vitro* findings were confirmed in an *in vivo* study where chick embryo retinas were treated for 48 h with CGS 21680. In this study, CGS 21680 was able to increase the expression of A_1_AR which was blocked by selective A_2A_AR antagonists, SCH 58261 and ZM 241385. Interestingly, under normal conditions, endogenous adenosine produced and released into the environment of the retina appears to control A_2A_AR-mediated expression of A_1_AR, since the treatment with ZM 241385 and SCH 58261 alone was capable of reducing the expression of A_1_AR [57]. These findings indicate that up-regulation of A_1_AR by long-term activation of A_2A_AR depends on classical AR signaling pathway.

A study done in rat hippocampus challenged with hypoxia showed internalization and desensitization of the A_1_AR, but without a reduction in the overall amount of these receptors [54]. Another study in C6 glioma cells subjected to hypoxia (2, 6 and 24 h) showed down-regulation of the A_1_AR and up-regulation of A_2A_AR receptor. This effect was shown to be dependent on the release of adenosine, since treatment with adenosine deaminase was able to block this effect. Peculiarly, the effect of the A_2A_AR expression induced by hypoxia was inhibited by A_1_AR antagonist, DPCPX, but not by the A_2A_AR antagonist, ZM 241385, indicating that the increased expression of the A_2A_AR receptor induced by hypoxia depends on the A_1_AR [58].

### 2.2. A_2A_AR

A_2A_AR desensitization is mediated by multiple, temporally distinct, agonist-dependent processes. Short-term agonist exposure induced a rapid desensitization of A_2A_AR-stimulated adenylyl cyclase activity which was associated with diminished receptor-G_s_ coupling, and agonist-stimulated phosphorylation of the A_2A_AR receptor protein. Longer agonist treatment, however, resulted in down-regulation in total receptor number and up-regulation of α-subunits of inhibitory G-protein [59]. The structural requirements necessary for the agonist-induced desensitization of A_2A_AR resides mainly in its carboxy-terminus. Palmer and Stiles [60] introduced various mutations in the 95 amino acid sequence from the carboxy-terminus of A_2A_AR which contains ten different phosphorylation sites and identified threonine-298 to be essential in agonist-induced receptor phosphorylation and short-term desensitization. However, it is not involved in long-term desensitization of A_2A_AR function. These findings confirm that short-term and long-term desensitization of A_2A_AR could be mediated by structurally distinct regions of the receptor protein and may involve different mechanisms.

GRKs play an important role in mediating agonist-induced phosphorylation and subsequent desensitization of GPCR action. Out of the different isoforms of GRKs, few of them have been shown to regulate A_2A_AR desensitization. NG108-15 mouse neuroblastoma/rat glioma cells over-expressing wild-type GRK-2 showed marked reduction in the adenylyl cyclase activity after acute A_2A_AR stimulation and enhanced sensitivity of A_2A_AR to desensitization. This phenomenon was found to be dependent on the levels of GRK-2. In cells expressing very high levels of GRK-2, low agonist concentration was sufficient to induce GRK-dependent desensitization [61]. This GRK-2-mediated A_2A_AR desensitization is reportedly inhibited by tumor necrosis factor (TNF)-α in human monocytoid THP-1 cells representing a novel cross-talk between TNF-α receptor and A_2A_AR. TNF-α treatment in THP-1 cells not only reduced the translocation of GRK-2 to the plasma membrane but also decreased GRK-2 association with the plasma membrane preventing A_2A_AR activity and enhancing receptor function [62]. Inhibitors of receptor internalization, such as hypertonic sucrose or concanavalin A did not affect agonist-stimulation of A_2A_AR or agonist-induced desensitization of receptor response in NG108-15 cells. However, incubation of these cells with sucrose or concanavalin A did affect the resensitization of A_2A_AR response following agonist removal [63].

### 2.3. A_2B_AR

Point mutation or deletion studies of rat A_2B_AR stably expressed in CHO cells revealed that a serine residue (Ser_329_), close to the carboxy-terminus, is critical for the rapid agonist-induced desensitization and internalization of the receptor [64]. A_2B_AR undergoes rapid agonist-induced desensitization and internalization in a GRK-2 and arrestin-dependent manner. Expression of a dominant negative mutant of GRK-2 in NG108-15 cells [65] or antisense-induced inhibition of non-visual arrestins (arrestin-2 and -3) in human embryonic kidney (HEK-293) cells [66] efficiently reduced the rate of agonist-induced desensitization and internalization of endogenous A_2B_AR. Recycling of A_2B_AR after agonist-induced endocytosis was also affected in cells with reduced arrestin levels. Interestingly, overexpression of arrestin-2 or arrestin-3 rescued A_2B_AR internalization and recycling of the receptor protein. Overexpression of arrestin-3, however, showed a significantly faster rate of recycling than arrestin-2, suggesting an isoform specific role of arrestin in regulating A_2B_AR trafficking [67]. In another study, A_2B_AR was shown to internalize in an arrestin- and dynamin-sensitive manner [68].

On the other hand, second messenger-dependent kinases, such as PKA and PKC, did not seem to be involved in agonist-mediated phosphorylation and subsequent desensitization of A_2B_AR [69]. Human astrocytoma cells which endogenously expressed A_2B_AR showed that TNFα, a pro-inflammatory cytokine, markedly reduced agonist-dependent receptor phosphorylation on threonine residues and attenuated agonist-mediated A_2B_AR desensitization [70]. TNFα-induced inhibition of A_2B_AR desensitization could result in prolonged A_2B_AR responsiveness and may contribute to the excessive astrocytic activation that occurs in neurodegenerative diseases.

In a recent study, it was proposed that A_2A_AR is involved in the surface expression of A_2B_AR in human embryonic kidney cell line, AD-293, transfected simultaneously with mouse A_2A_AR and A_2B_AR. It was found that newly synthesized A_2B_AR is retained in the endoplasmic reticulum (ER) and is eventually targeted for degradation by the proteosomes. Inhibition of proteosome activity was not sufficient to enhance maturation and surface expression of the receptor. Furthermore, it was shown that co-transfection of A_2A_AR with A_2B_AR enhanced surface expression of A_2B_AR through F(X)_6_LL motif in A_2A_AR carboxy-terminus. These findings support the notion that A_2A_AR and A_2B_AR form heterodimer complexes for trafficking and function [71].

### 2.4. A_3_AR

Agonist occupation of A_3_AR results in rapid desensitization of receptor function as a result of phosphorylation of the receptor protein by members of the family of GRKs [72,73]. To identify the amino acid residue important for A_3_AR phosphorylation, Palmer and Stiles [74] demonstrated that a triple mutant, Thr^307^/Ala^307^, Thr^318^/Ala^318^ and Thr^319^/Ala^319^ within the carboxy-terminus, showed marked reduction in agonist-stimulated phosphorylation and desensitization of rat A_3_AR. Individual mutations of each residue showed that Thr^318^ and Thr^319^ are the two major sites for phosphorylation where Thr^318^ appeared to be necessary to observe phosphorylation at Thr^319^, but not vice versa. Moreover, mutation of two palmitoylation sites within the carboxy-terminus, Cys^302^ and Cys^305^ of rat A_3_AR which controls the GRK phosphorylation sites, displayed a significant level of basal phosphorylation even in the absence of an agonist. This suggests an important regulatory role of these palmitoylation sites in receptor desensitization.

Receptor kinetics of A_3_AR was studied by Trincavelli *et al.* [75] in human astrocytoma cells. Short-term exposure to the agonist 2-chloro-*N*6-(3-iodobenzyl)-*N*-methyl-5′-carbamoyladenosine (Cl-IBMECA) caused rapid receptor desensitization followed by internalization within 15 and 30 min, respectively. Agonist removal resulted in recycling of the A_3_ARs to the cell surface within 120 min. Long-term exposure (1–24 h) resulted in marked receptor down-regulation and the restoration of receptor levels associated with recovery of receptor function was also slow (24 h). MAPK was shown to regulate agonist-induced phosphorylation of A_3_AR where its stimulation mediated activation of ERK1/2 within 5 min of agonist exposure in CHO cells stably expressing A_3_AR. Treatment with PD98059, a well-characterized MAPK kinase inhibitor, showed impaired receptor phosphorylation, desensitization and internalization by inhibiting GRK-2 translocation from cytosol to the plasma membrane. These data suggests that A_3_AR activation is regulated by ERK1/2 in a feedback mechanism which controls GRK-2 activity and prevents receptor phosphorylation [76]. These findings could explain the dual and opposite role of A_3_AR in neuroprotection and neurodegenerative diseases.

## 3. NF-κB Regulation of A_1_AR and A_2A_AR Expression

Previous studies from our laboratories indicated that the expression of the A_1_AR is positively regulated by oxidative stress. This observation was made from studying the action of cisplatin, a chemotherapeutic agent, on the levels of A_1_AR in the chinchilla cochlea [77]. It was shown that cisplatin, via an oxidative stress pathway, induces A_1_AR in the cochlea which could serve as a last-ditch effort to rescue the cells in the inner ear from apoptosis. More detailed studies in DDT_1_-MF2 cells showed that cisplatin-induced A_1_AR expression resulted from activation of NF-κB by reactive oxygen species (ROS) produced by cisplatin. More importantly, an NF-κB consensus sequence was located 623 base pairs upstream of the start site of the human promoter A construct [78]. Such a mechanism of A_1_AR induction could help to precondition the cell or tissue to subsequent episodes of oxidative stress. For example, noise exposure, which increases ROS in the chinchilla cochlea (via NADPH oxidases), increases A_1_AR in the inner ear [79] while osmotic diuresis increases A_1_AR in the kidneys by a similar mechanism [80]. A similar mechanism could explain the induction of the A_1_AR and A_3_AR in the gut in a rabbit model of ileitis [81] and in the brain after the induction of cerebral ischemia in rats [82].

We have previously shown that activation of NF-κB is also a key regulator of A_2A_AR expression. Treatment of PC12 cells with nerve growth factor (NGF) significantly reduced *A_2A_AR* gene expression by an approximately three 3-fold within three days [83]. This effect was associated with a rapid activation of NF-κB via the low affinity p75 NGF receptor and was mimicked by other activators of NF-κB (e.g., ceramide, H_2_O_2_) [84]. These results are consistent with findings of NF-κB consensus sites present in the *A_2A_AR* gene promoter [85]. However, other mechanisms could be invoked to explain NGF regulation of A_2A_AR. These include ERK and stress-activated protein kinase/c-Jun *N*-terminal kinase (JNK) [86].

To evaluate the significance of NF-κB in the regulation of the A_1_AR expression, we examined the expression of ARs in brain tissues from mice with deletion of the gene for the p50 subunit of NF-κB. This gene knockout (KO) renders these mice immune deficient, but they are viable and able to reproduce [87]. These mice showed reduced levels of A_1_AR in brain cortical plasma membranes, compared to their wild type counterparts. Interestingly, the levels of the G proteins alpha subunits (Gα_i3_) were also significantly reduced in the p50 KO mice, but the levels of Gα_i1_ were unchanged. The deficit in A_1_AR/G_i_ protein expression was associated with increased neuronal apoptosis [88]. These findings suggest that NF-κB tonically regulates neuronal A_1_AR expression and survival.

Based on the observation that the A_2A_AR is negatively regulated by NF-κB, we were interested in the expression of this receptor in p50 KO mice. We observed higher expression of the A_2A_AR in striatal tissues from p50 KO mice, compared to wild type mice. As anticipated, less A_1_AR mRNA and protein was detected in the striatal tissues in p50 KO mice as compared with F2 mice [89]. These studies suggest that absence of the NF-κB p50 subunit leads to dysregulation of ARs in the striatum, as observed for the A_1_AR in other brain regions. Overall, these studies suggest an essential role of the NF-κB p50 subunit in the regulation of A_1_ and A_2A_AR expression in the brain.

Alterations in A_2A_AR in striatum could alter locomotor activity, since this receptor exhibits inhibitory actions on dopamine D_2_ receptor (D_2_R). The wild type and p50 KO mice did not show any difference in basal locomotor activity, but the p50 KO mice showed a hypersensitivity to caffeine-induced locomotor activity evaluated during the dark phase (when mice are normally active) [90]. The p50 KO mice also demonstrated increased sensitivity to intraperitoneal injections of SCH 58261, an A_2A_AR antagonist [90] but not to the selective A_1_AR antagonist, DPCPX. These data suggest that the increase in A_2A_AR in p50 KO mice provides larger striatal target (A_2A_AR) for inhibition by caffeine, resulting in behavioral hypersensitivity.

## 4. Adenosine Receptor Oligomers

GPCRs are usually treated as individual units responsible for a specific signaling pathway. However, it is now known that these receptors can form complex structures with each other through physical links in its protein structure. When binding occurs between the same GPCRs, it forms a homodimer. On the other hand, when the link is between different receptors, it is a heterodimer. The term heteromer is currently defined as a macromolecular complex consisting of two or more functional receptors with different biochemical characteristics of individual receptors [91]. Moreover, the activation of a member of the heteromeric complex alters the binding properties of other receptor(s) present in the complex [91].

In this regard, a variety of papers in the literature indicate that ARs are capable of forming homodimers with each other and heterodimers with other GPCRs. One of the earliest evidence of the formation of homodimers of A_1_ARs has been demonstrated in intact tissue in the cortex of different species by immunoblotting and coimmunoprecipitation techniques [92]. Using immunogold electron microscopy, the authors confirmed the presence of homodimers. The presence of A_1_AR homodimers has also been demonstrated in hippocampal pyramidal neurons and Purkinje cells of the cerebellum [93]. Recently it was shown that HEK-293T cells transfected with the cDNA for the A_1_AR express receptor homodimers which promoted phosphorylation of ERK. The expression of A_1_AR homodimers in the cortex could explain the biphasic effects of the small and high doses of caffeine on motor activity [94]. Homodimers of A_2A_AR receptors have been demonstrated in HeLa and HEK-293T cells co-transfected with different constructs of A_2A_AR receptor. Such cells produce functional receptors expressed on the cell surface and the formation of the homodimer apparently was not dependent on the activity of the receptor, since treatment with the A_2A_AR agonist CGS 21680 did not alter the bioluminescence resonance energy transfer (BRET) signal detected. Therefore, these data suggest that A_2A_AR homodimers are constitutively expressed in native tissues and cultured cell lines [95]. However, it is yet unclear how these homodimeric A_1_ARs differ functionally from their monomeric counterparts.

As mentioned above, ARs can form heterodimers with each other. Ciruela and coworkers [96] showed that co-transfection of the A_1_ and A_2A_AR receptor cDNAs in HEK-293T cells led to the formation of A_1_/A_2A_AR heterodimers on the cell surface. Interestingly, activation of the A_2A_AR by CGS 21680 in these cells reduced the affinity of the A_1_AR receptor to a selective radioligand. However, activation of A_1_AR by the A_1_AR agonist, R-PIA, did not produce a reciprocal change in the affinity of the A_2A_AR [96]. The ability of the A_2A_AR to reduce the affinity of the A_1_AR was confirmed in experiments that measured the levels of intracellular calcium. Incubation of the A_1_/A_2A_AR-transfected HEK-293T cells with R-PIA increased accumulation of intracellular calcium, which was drastically reduced when these cells were pretreated with CGS 21680 [96]. *In vivo* studies in rat striatum demonstrated co-localization of A_1_ and A_2A_ARs in the extrasynaptic terminals of axons as well as in the presynaptic active zone of excitatory glutamatergic neurons, an indication that these receptors could be forming heterodimers. Using synaptosomes enriched in striatal glutamatergic nerve terminals, the investigators showed that the A_1_AR inhibited K^+^-evoked glutamate release was abolished by A_2A_AR activation [96]. In cultures of rat astrocytes, Christovão-Ferreira *et al.* [97] showed that A_1_ and A_2A_AR form heterodimers. In these cells, activation of the A_1_AR inhibited GABA uptake while activation of the A_2A_AR increased uptake. Surprisingly, the effect of the A_1_AR was blocked not only by the selective antagonist DPCPX, but also by the SCH 58261, a selective A_2A_AR antagonist. In turn, the effect of A_2A_AR on GABA uptake was blocked by SCH 58261 and DPCPX, indicating cross antagonism and/or a physical interaction between these receptors [97]. Both receptors were shown to be internalized together when exposed to A_1_ and A_2A_AR agonists separately. Interestingly, the differential responses of A_1_ and A_2A_AR agonists on GABA uptake involved activation of the G_i/0_ and G_s_ protein, respectively [97]. Thus, the presence of A_1_–A_2A_AR heterodimers in the central nervous system could increase the complexity by which these two receptors regulate neuronal excitability. However, the advantage of A_1_–A_2A_AR heterodimers regulation of GABA uptake over the effects of the individual ARs is unclear.

Evidence from the literature suggests that an interaction exists between adenosine receptors and adrenergic receptors. For example, activation of the A_1_AR in myocardium attenuates β_1_-induced cyclic AMP formation and PKA enzymatic activity [98]. In cardiomyocytes, activation of the A_1_AR receptor by selective agonist CCPA translocates protein kinase C ɛ (PKCɛ) to the membrane. This effect was attenuated by isoproterenol, a non-selective β-adrenergic receptor agonist or forskolin, an activator of adenylyl cyclase [99]. These results are suggestive of an interaction between A_1_AR and the β-adrenergic receptors. HEK-293T cells co-transfected with A_1_AR, β_1_ and β_2_ adrenergic receptors show A_1_AR/β_1_AR or A_1_AR/β_2_AR heterodimers which enhance the phosphorylation of ERK induced by isoproterenol or CCPA, as compared to cells transfected with only one type of receptor. In addition, the inhibitory effect of A_1_AR on cyclic AMP production mediated by CCPA was abolished in cells expressing both heterodimers. Analysis of samples of human heart tissue showed the formation of these heterodimers [100]. Thus, the data suggest that A_1_AR and adrenergic receptors are able to form heterodimers with different properties compared to the generally accepted pharmacological and functional properties of individual receptors.

The principal source of production of adenosine into the extracellular environment occurs through metabolism of ATP [11]. Thus, the amount of ATP released into the extracellular medium directly determines the activity of ARs. However, ATP itself influences the activity of its receptors, which are subdivided into P2X, P2Y, P2U and P2Z subtypes. All members of the P2Y family of receptors are G protein-coupled. Studies indicate that the P2Y1 receptor is able to form heterodimers with the A_1_AR *in vitro*. These heterodimers were less effective in inhibiting forskolin-stimulated cyclic AMP formation than the native A_1_AR [101]. Imaging by BRET in HEK-293T cells confirms the formation of P2Y1/A_1_AR heterodimers [102]. Immunostaining of rat cortical neurons showed that both receptors co-localize in the cell body and dendritic regions. In rat cortex slices these receptors are shown to be present together in cortical, hippocampal, and cerebellar neurons, mainly in the soma and dendrites, indicating that these receptors could be form heterodimers. Moreover, immunoprecipitation analysis and electron microscopy of these specific brain regions have reported an interaction between the A_1_AR and P2Y1 receptors [93,103].

A_2A_ARs are highly expressed in the striatum [5], a region that also has an abundance of cannabinoid (CB)-1 receptors [104]. CB1 receptors are coupled to G_i/o_ proteins and belong to a subtype of receptor for exogenous cannabinoids and endocannabinoids. The latter are represented by 2-arachidonoylglycerol (2AG) and *N*-arachidonoylethanolamine (anandamide), which differ from the majority of neurotransmitters because they are produced in the post-synaptic neuron and released by retrograde transport to the pre-synaptic neuron to perform its functions [104]. Immunofluorescence studies in coronal sections of rat striatum revealed a co-localization between A_2A_AR and CB1 receptors in striatal neurons. Subsequent immunoprecipitation experiments confirmed A_2A_AR-CB1 in striatal cells [105]. BRET assays in HEK-293 cells, co-transfected with A_2A_AR and CB1 receptors, revealed the formation of heterodimers on the cell surface [105,106]. *In vitro* studies in human neuroblastoma SH-SY5Y cells constitutively expressing A_2A_AR and CB1 receptors revealed a functional cross-talk between these receptors. When these cells were treated as selective CB1 receptor agonist, arachidonyl-2′-chloroethylamide (ACEA), the accumulation of cyclic AMP induced by forskolin was reduced. However, in the presence of the selective A_2A_AR antagonist, ZM 241385, the decrease in cyclic AMP by ACEA was reduced. These data suggest that effective coupling of the CB1 receptor to G_i_ protein requires prior or simultaneous activation of the A_2A_AR [105]. Moreover, CGS 21680 increase cyclic AMP production in SH-SY5Y cells, which is blocked by either ZM 241385 or ACEA. Thus, activation of A_2A_AR, leading to the production of cyclic AMP, required concurrent activation of the CB1 receptor [105]. Taken together, the data described above suggest that the A_2A_AR can heterodimerize with the CB1 receptor, which controls how each of these receptors responds to their specific agonists [107,108].

The best studied heterodimer of ARs are with dopamine receptors (DRs). Using double immunofluorescence assays, Hillion *et al.* [109] demonstrated a high degree of co-localization between A_2A_AR and D_2_R in the cell membranes of SH-SY5Y cells stably transfected with the human D_2_R and in cultures of striatal rat neurons. The existence of A_2A_AR-D_2_R heterodimeric complex was confirmed by co-immunoprecipitation assays. Interestingly, co-administration of A_2A_AR and D_2_R agonists to SH-SY5Y cells transfected with D_2_R cDNA resulted in co-aggregation, co-internalization and co-desensitization of the A_2A_AR and D_2_R [109]. A_2A_AR-D_2_R heterodimers were also revealed in HEK-293T cells co-transfected with the cDNAs encoding these receptors by fluorescence resonance energy transfer (FRET) and co-immunoprecipitation studies [110–112]. In HEK-293T cells co-transfected with A_2A_AR and D_2_R, Borroto-Escuela *et al.* [110] demonstrated that quinpirole (D_2_R agonist) induced internalization of the receptor heterodimer complex, which was facilitated by CGS 21680 (A_2A_AR agonist). Furthermore, internalization of the complex was shown to be dependent on β-arrestin2 and Akt [110]. Other data also show that the A_2A_AR-D_2_R complex can associate with the CB1 receptor [106] or the metabotropic glutamate receptor, mGluR5 [113] to form a trimeric complex. Studies have also shown formation of A_1_AR-D_1_R heterodimers. For example, co-transfection of mouse fibroblasts with A_1_AR and D_1_R cDNAs led to the formation of heterodimers. These heterodimers disappeared when these cells are pretreated with the SKF 38393, a D_1_R agonist. Using immunofluorescence labeling and confocal microscopy detection, co-localization or A_1_AR and D_1_R were observed in fibroblast cultures and cortical neurons [114]. Immunoprecipitation studies of tissues obtained from the rat nucleus accumbens demonstrated the presence of A_1_AR-D_1_R heterodimers in this brain region. Interestingly, the levels of heterodimers were significantly reduced in rats previously treated with cocaine, suggesting that activation of the D_1_R component of the heterodimer complex triggers its dissolution [115]. The presence of heterodimers between ARs and DRs in cells of the central nervous system would suggest some added functional roles of these complexes other than those mediated by activation of the individual receptors. In the striatum, one can envision that the AR-DR heterodimers could help to fine tune the intracellular signaling processes emanating from extracellular adenosine and dopamine or therapeutic agents directed to these receptors. As such, a better appreciation of AR-DR heterodimers could provide better insights to the treatment of diseases involving the striatum, such as Parkinson’s and Huntington’s disease [116–118].

## 5. Adenosine Receptors in the Control of Sleep

Adenosine has been shown to serve as a sleep promoting factor. The levels of this nucleoside in the basal forebrain increase during prolonged wakefulness and resolves during sleep [119]. Furthermore, individuals expressing an adenosine deaminase polymorphism, which increases adenosine, show deeper sleep and higher slow-wave activity (SWA) [120]. Infusion of adenosine in the basal forebrains increases sleep in rats [121] and cats [122]. Similarly, inhibition of adenosine intracellular uptake by inhibition of its equilibrative transporter produces electrophysiological and behavioral parameters similar to those produced by sleep deprivation [123]. Several pieces of evidence support a role of the A_1_AR (localized to the basal forebrain) in mediating the sleep promoting actions of adenosine. For example, systemic or cerebroventricular administration of A_1_AR agonists to rats led to increased sleep drive [124,125]. Administration of selective A_1_AR antagonist or A_1_AR antisense oligonucleotides (to reduce A_1_AR expression) increased wakefulness in rats. Interestingly, an increase in A_1_AR density in the basal forebrain was observed following sleep deprivation, which could contribute to the subsequent sleep rebound [124]. It is believed that the source of the excessive levels of adenosine during wakefulness derives from astrocytes and is released via soluble *N*-ethylmaleimide-sensitive factor attachment protein receptor (SNARE)-dependent gliotransmission [126]. Mice expressing an astrocyte-specific dominant negative SNARE protein show reduced SWA, compared with their wild-type counterparts [126]. This deficit could be produced in wild-type mice by intracerebroventricular infusion of cyclopentyltheophylline. In addition, these investigators observed that mice expressing the dominant negative SNARE protein had a lower level of memory deficit induced by sleep deprivation as compared to their wild-type littermates. This suggests a role of adenosine (produced during sleep deprivation) in mediating the memory deficits [126].

Recent studies using A_1_AR and A_2A_AR KO mice, clearly demonstrate a role of the A_2A_AR (not the A_1_AR) in mediating both the sleep suppressing and arousal actions of caffeine [127]. However, these investigators showed that the A_1_AR in the tuberomammillary nucleus mediates non-rapid eye movement (non-REM) sleep by inhibiting histamine release [128].

Since the A_1_ and A_2A_ARs are induced by NF-κB in cell cultures [78,84], we examined the expression of these receptors in the cortex and striatal tissues of mice with a global deletion in the p50 subunit of NF-κB. As anticipated, these mice showed reduced expression of the A_1_AR in different regions of the brain and significant elevations in A_2A_AR in the striatum, suggesting that NF-κB activation controls the normal expression of these receptors. We also evaluated the sleep patterns of these mice to determine whether the modest decrease in A_1_AR would produce any changes. These mice exhibited more slow wave and REM sleep under normal conditions than their wild type counterparts (B6129PF2/J strain) [129]. This finding was surprising, based on the purported role of the A_1_AR in regulating sleep (see above) in rats. Accordingly, a lower level of A_1_AR expression should lead to reduced sleep duration. The p50 KO mice also demonstrate a more rapid recovery to normal sleep patterns following sleep deprivation. These data suggest dissociation between A_1_AR expression and normal sleep patterns in mice and in mediating the homeostatic drive following sleep deprivation.

**Figure 1.**
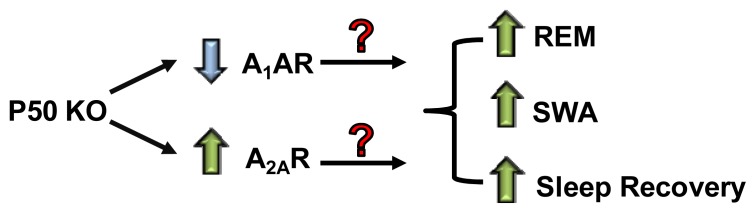
Proposed model of regulation of sleep by adenosine receptors in p50 KO mice. These mice express lower levels of A_1_AR in the cortex and striatum but higher expression of the A_2A_AR. P50 KO mice also demonstrate increased REM and SWA sleep and increased rate of sleep recovery following sleep deprivation. (?) indicates question as to which receptors to attribute the differences in sleep pattern to.

The p50 KO mice also showed increased expression of the A_2A_AR in the striatum [89] and possibly in other brain regions. The increased striatal expression of A_2A_AR in the KO mice could account for their increased responsiveness to caffeine [89]. Since the A_2A_AR has been implicated in mediating the increased sleep propensity to adenosine and arousal actions of caffeine [127], it is tempting to speculate that the increase in cortical A_2A_AR could explain the increases in slow wave and REM sleep observed in the P50 KO mice, in addition to their rapid recovery following sleep deprivation (see Figure 1).

## 6. Adenosine Receptor and Protection against Hearing Loss

**Figure 2.**
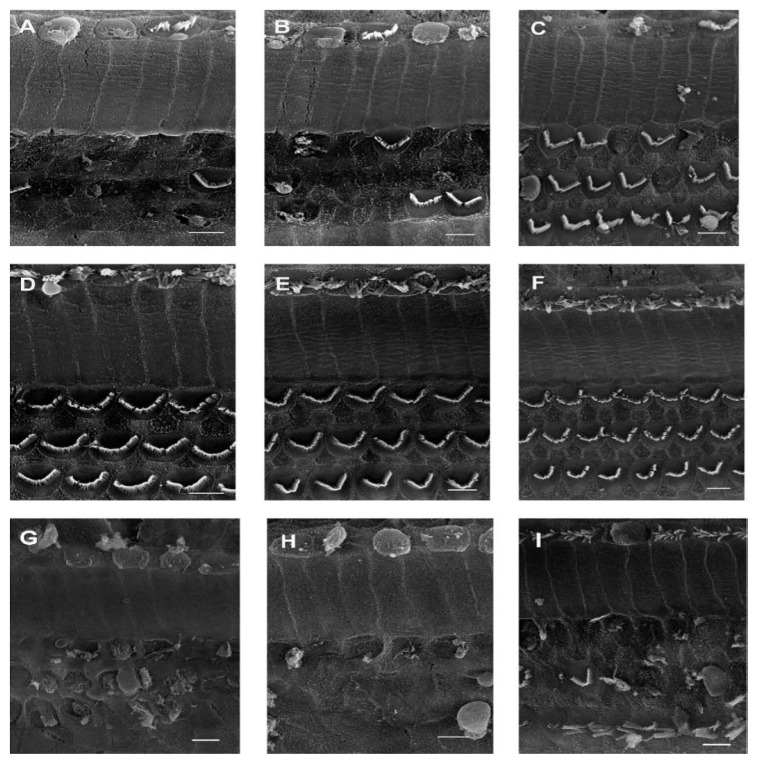
Protection of rat cochlear outer hair cells from cisplatin-induced damage by A_1_AR agonist, R-PIA. Rats were pre-treated with R-PIA (10 μL of a 10 μM solution added to the round window). The remaining liquid was removed after 1 h and rats were administered cisplatin by intraperitoneal injections (16 mg/kg). Panels **A**–**C** are electron micrographs of the organ of Corti from rats treated with cisplatin and represent the hook, basal turn and the middle turn, respectively; Panels **D**–**F** shows the effect of pre-treatment with R-PIA for the respective regions; Panels **G**–**I** show the effect of R-PIA in presence of DPCPX, an A_1_AR antagonist, on cisplatin-induced damage. The inner hair cells are the three rows of cells with the “V” shaped stereociliary bundles. The inner hair cells are barely visible on the top of each micrograph. The data which was confirmed from different cochlear preparations support a protective role of the A_1_AR against cisplatin ototoxicity. (Reprinted with permission from [134], Copyright 2004 Elsevier). Scale bar = 5 μm.

Early studies indicate that adenosine could modulate afferent neurotransmission in hair cell of the frog labyrinth system, a model system for studying hair cell [130]. These investigators showed that adenosine and adenosine uptake inhibitors reduced, while theophylline and adenosine deaminase increased, firing of hair cell afferents [130]. A subsequent study by Dulon *et al.* [131] demonstrated that application of adenosine to mammalian inner hair cells increased intracellular Ca^2+^ levels. Direct demonstration of A_1_AR in the rat cochlea, using several biochemical techniques, was later provided [132]. These receptors did not alter sound-evoked endocochlear potentials but activated antioxidant enzymes in the cochlea, thereby reducing the basal levels of malondialdehyde, a marker of lipid peroxidation and cell damage [77]. Activation of the A_1_AR contributes to the otoprotective actions of the adenosine analog, R-PIA, against cisplatin ototoxicity ([133,134] see Figure 2) and noise-induced hearing loss [135]. An interesting aspect of the A_1_AR in the cochlea is that it could be induced by oxidative derived from certain chemotherapeutic agents (such as cisplatin and aminoglycoside antibiotics) and noise. Induction of A_1_AR appears to be dependent on the activation of NF-κB by ROS which enhance transcriptional activity of the *A_1_AR* gene [78]. We propose that such feedback regulation could increase the cytoprotective activity of the A_1_AR in response to oxidative stress.

The NADPH oxidase system appears to contribute significantly to cochlear ROS produced by noise trauma and cisplatin. Specifically, the NOX3 isoform of NADPH oxidase is localized primarily to the cochlea and mediates cisplatin ototoxicity [136]. As such, knockdown of NOX3 by trans-tympanic administration of NOX3 siRNA, protected against cisplatin ototoxicity [137]. ROS can increase DNA damage, and modify membrane lipids, cytosolic proteins and receptors on the cell surface [138]. Increased lipid peroxides appear to induce cochlear damage following noise exposure [139]. This explains the protective actions of antioxidants to treat hearing loss induced by noise or therapeutic agents [140]. However, the use of these agents to treat cisplatin-induced ototoxicity is limited by the potential interaction of the antioxidants and cisplatin to reduce its chemotherapeutic efficacy.

Recent studies have revealed another target of ROS, induction of inflammation in the cochlea. ROS can increase inflammatory processes in the cochlea by activating NF-κB [78,79]. NF-κB is a known mediator of inflammatory processes in the body [141]. More recently, we have shown that oxidative stress in the cochlea can contribute to the inflammatory process by activating signal transducer and activator of transcription 1 (STAT1) transcription factor [142]. In addition, STAT1 is able to couple the activation of transient receptor potential vanilloid receptor (TRPV)-1 in the cochlea to the induction of inflammation [143]. Accordingly, down-regulation of STAT1 ameliorates cisplatin-induced ototoxicity in the rats [142]. It is possible that the otoprotective actions of the A_1_AR against cisplatin ototoxicity involve inhibition of NF-κB and STAT1 transcription factors.

The cochlea expresses three subtypes of the ARs [144]. The A_1_ARs are expressed in inner hair cells, Deiters cells and spiral ganglion neurons. The A_2A_AR are present in the inner hair cells, Deiters cells, spiral ligament and spiral ganglion. However, the A_3_AR appear to have a more wide-spread expression. While it is clear that the activation of the A_1_AR confers otoprotection, the role of the A_3_AR is less clear. On the other hand, activation of the A_2A_AR appears to exacerbate cisplatin-induced ototoxicity [134]. As such, potentially useful AR drugs to treat ototoxicity could include agonists of the A_1_AR and antagonists of the A_2A_AR.

## 7. Adenosine Receptors in Cancer

The expression levels and function of A_1_AR in different cancer types is highly debatable. Panjehpour *et al.* [145] using semi-quantitative RT-PCR, reported that there was no significant difference between the expression levels of A_1_AR in non-neoplastic normal tissues and human breast tumor tissues. However, in another study, Mirza *et al.* [146] found high levels of A_1_AR in all breast tumor cell lines as well as in 15 of 24 human primary breast cancer tissues compared to normal mammary epithelial cells and matched normal breast tissues, respectively. In this study, knockdown of A_1_AR using siRNA attenuated MDA-MB-468 human breast tumor cell growth and proliferation by impairing the G_1_ checkpoint in the cell cycle. High expression of A_1_AR was also found in human colorectal adenocarcinoma [147], human leukemia Jurkat T cells [148] and human melanoma A375 cell lines [149]. Increased A_1_AR was also observed in the peritumoral regions of F98 glioblastoma cells [150]. The source of increased A_1_AR expression was found to be activated astrocytes and microglia which suppressed growth of glioblastoma upon A_1_AR activation. Despite its role in tumorigenesis, few studies have shown A_1_AR to have anti-tumor effects. In CW2 human colon cancer cells, A_1_AR activation increased apoptosis by activating caspases [151]. Anti-proliferative effects of A_1_AR were also reported in LoVo human metastatic colon cancer cells [152] and Sertoli-like TM4 cells [153].

A_2A_AR are found to be over-expressed in several cancer cell lines such as MCF-7 human breast cancer cells [154], Jurkat T-cell leukemia [148], A375 melanoma cells [149], U87MG human glioblastoma cells [155], HT29 and DLD-1 colon carcinoma cells [156], SH-SY5Y neuroblastoma cells [157], and non-small cell lung cancer cells [158] where it is shown to affect cell proliferation, apoptosis, angiogenesis and anti-tumor immunity. Activation of the A_2A_AR by CGS 21680 was shown to stimulate cell proliferation in MCF-7 breast cancer cells [154]. A_2A_AR also induced angiogenesis by promoting endothelial cell proliferation, migration and tube formation which is an important hallmark of cancer [159,160]. In another study, A_2A_AR was reported to protect the tumor by inhibiting anti-tumor T cells and A_2A_AR antagonist facilitated CD8+ T cell-mediated retardation of tumor growth [161]. These findings warrant the use of A_2A_AR antagonists to inhibit tumor growth and angiogenesis. In contrast, A_2A_AR activation in A375 melanoma cells reduced cell viability and cell clone formation [162], and activated caspases to induce apoptosis in Caco-2 human colon cancer cells [163].

The role of A_2B_AR in cancer can be broadly classified as pro-angiogenic. A_2B_AR activation in human retinal endothelial cells stimulated neovascularization through production of vascular endothelial growth factor (VEGF) [164]. A_2B_AR knockout mice, administered with Lewis lung carcinoma cells, demonstrated reduced tumor growth and longer survival times, compared to wild type control mice. It was suggested that tumor cells promote their growth by exploiting A_2B_AR-dependent regulation of VEGF in host immune cells [165]. In addition, knockdown of A_2B_AR by siRNA inhibited the release of pro-angiogenic growth factor, interleukin-8, in A375 human melanoma cells treated with chemotherapeutic agents like etoposide and doxorubicin [166]. The A_2B_AR promoter region contains a functional binding site for hypoxia-inducible factor (HIF), which leads to the induction of A_2B_AR under hypoxic conditions [167]. A_2B_AR is also over-expressed in colorectal carcinoma cells grown under hypoxic state, the inhibition of which significantly attenuated cell proliferation [168].

**Figure 3.**
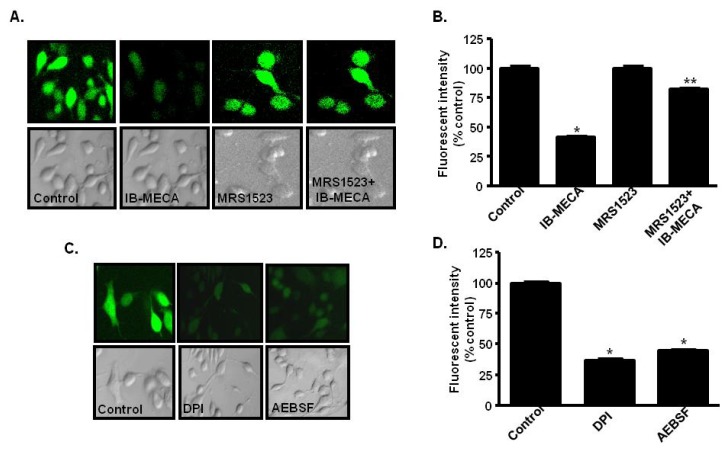
Activation of A_3_AR suppresses ROS generation in AT6.1 prostate cancer cells. Cells were pretreated with the A_3_AR agonist, IB-MECA, the antagonist, MRS1523, or a combination of IB-MECA + MRS1523. ROS was determined by H_2_DCFDA fluorescence. IB-MECA significantly reduced fluorescence in these cells (**A**,**B**) which was reversed by MRS1523, indicating a role of the A_3_AR in this process. Similar effects were obtained with DPI and AEBS*F*, known inhibitors of NADPH oxidase (**C**,**D**). Experiments were replicated four times. Histograms represent the mean ± SEM. Asterisks (*) and (**) indicates statistically significant difference (*p* < 0.05) from vehicle and IB-MECA treatments, respectively. (Reprinted with permission from [169], Copyright 2009 Neoplasia Press, Inc.).

A_3_ARs are over-expressed in different types of cancer cells, such as PC-3MM human prostate cancer cells [169], colon and breast carcinoma tissues [170], A375 human melanoma cells [146], U87MG human glioblastoma cells [155,171], HL60 human promyelocytic leukemia cells [172], and hepatocellular carcinoma [173] compared to their normal counterparts, suggesting that these receptors could serve as potential molecular markers of these cancers. A role of the A_3_AR in mediating anti-tumor actions has been demonstrated in *in vitro* and *in vivo* models. For example, A_3_AR agonists inhibit the growth of melanoma [174], colon [175], leukemia [172] and prostate cancers [169,176] in animal models. A_3_AR agonist also inhibited liver metastasis in mice inoculated with human HCT-116 or murine CT-26 colon carcinoma cells [175]. The anti-tumor action of A_3_AR agonists could be explained by an increase in natural killer (NK) cell activity, which promotes killing of tumor cells [177]. In prostate cancer cells, Jajoo *et al.* [169] demonstrated that activation of the A_3_AR led to suppression of the high levels of ROS generated by these cells by inhibiting NADPH oxidases (Figure 3). Interestingly, activation of this receptor reduced ERK1/2 activity which normally controls the phoshorylation and activity of the p47^phox^ subunit of this enzyme.

## 8. Conclusions

As described above, adenosine and ARs play a dynamic role in regulating normal cell physiology and also act as modulators in disease processes. A better understanding of the functions of these receptors, especially the newly identified receptor homomers and heteromers, could stimulate development of new therapies for the treatment of diseases.
